# Recurrent vasovagal syncope following successful cardioneuroablation

**DOI:** 10.1016/j.hrcr.2022.04.001

**Published:** 2022-04-06

**Authors:** Clinton J. Thurber, Davis R. Sneider, William H. Sauer, Sunil Kapur

**Affiliations:** ∗ Brigham and Women’s Hospital, Boston, Massachusetts; † Abbott Laboratories, Abbott Park, Illinois

**Keywords:** Vasovagal syncope, Cardioneuroablation, Ganglionated plexuses, Catheter ablation, Recurrent syncope

## Introduction

Vasovagal syncope (VVS) is a common phenomenon, with upwards of 30%–45% of the population having suffered 1 VVS episode by the age of 60.^1^^,^^2^ It is a pathology that has frustrated many patients and providers in its suboptimal treatment response rate, and that has enacted an outsized economic impact on western societies.^3^ VVS subtypes are classified in 3 broad categories: a pure cardioinhibitory type (with and without asystole); a pure vasodepressor type, in which hypotension occurs without a significant decrease in heart rate (HR); and a mixed vasodepressor and cardioinhibitory type. Given the prevalence of treatment failure in VVS, combined with the fraught pursuit of permanent pacing in a relatively young patient population, minimally invasive denervation has been pioneered in recent years via radiofrequency (RF) cardioneuroablation (CNA).^4^, ^5^, ^6^, ^7^, ^8^, ^9^, ^10^, ^11^, ^12^ However, while recurrent syncope following CNA has been described, there have not been reports of recurrence coinciding with objective data reflecting the return of parasympathetic autonomic function. Herein we describe such a case, documenting cardioinhibitory VVS recurrence post-CNA with the use of an implantable loop recorder (ILR).

### Case report

**Figure 1:**
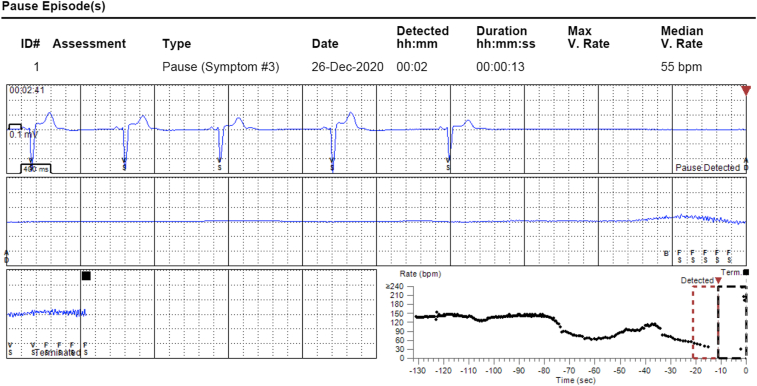
Preprocedural syncopal event. The patient’s implantable loop recorder recorded gradual sinus slowing followed by sinus arrest with asystole, causing syncope.

A 20-year-old woman with a prior medical history significant for mild iron deficiency anemia, frequent nausea, and multiple syncopal episodes was referred to the cardiac arrhythmia clinic for evaluation of recurrent syncope. Her family history is significant for cardioinhibitory-type VVS in her mother, successfully treated with permanent pacing. The patient described syncopal spells occurring approximately once per year since she was in grade school, becoming progressively more frequent and culminating in weekly syncope over the preceding 4 months. The most recent event leading to her referral resulted in a concussion and large thigh hematoma. Her physical exam and laboratory studies were unremarkable. Electrocardiogram revealed sinus bradycardia at a rate of 54 beats per minute (BPM), PR interval 138 ms, QTc interval 396 ms, and QRS interval 86 ms. Echocardiography exhibited a structurally normal heart. She underwent ILR placement, after which she suffered a syncopal event, which correlated with gradual sinus slowing followed by sinus arrest and asystole (Figure 1). On her follow-up visit, management options were discussed at length, including conservative measures such as hydration, compression stockings, and isometrics; pharmacologic intervention; and invasive options to include permanent pacemaker implantation or CNA. After lengthy discussion, she felt that conservative measures had not helped prevent her episodes in the past. Further, she expressed an aversion to additional medications and wished to pursue CNA prior to committing to permanent pacemaker implantation.

The patient presented to the procedural suite in a fasting state. General anesthesia was administered, and diagnostic catheters were employed to measure baseline conduction intervals, which were all within normal limits. Atropine 0.4 mg was then administered intravenously, and the HR response increased from a baseline rhythm of sinus bradycardia at a rate of 58 BPM to sinus tachycardia at a rate of 105 BPM. A plan for additional atropine titration to achieve a satisfactory HR response was not required. This higher HR gradually decreased over the subsequent hour during mapping and ablation, but remained elevated and above baseline throughout the procedure.

A high-definition mapping catheter (Advisor HD Grid Mapping Catheter; Abbott Medical, Plymouth, MN) was then advanced into the right atrium, and fractionation mapping was performed based on 2 primary variables: electrogram (EGM) width and refractory interval. EGM width describes the minimum EGM time (in milliseconds) to qualify for annotation, while the refractory interval dictates the minimum time required to transpire between annotations. In the present case, the EGM width was set to 5 ms and the refractory interval to 15 ms. Fractionated sites largely correlated with expected anatomical ganglionated plexus (GP) locations, and these were then assessed with high-frequency stimulation (HFS) to elucidate the site-specific contribution to overall vagal tone on the heart. Drive trains of 20 Hz, 1–20 mV amplitude, and 2.0 ms pulse width impulses were delivered for a duration of 5 seconds. Responses were variable, and included sinus rhythm, 1–3 junctional beats followed by sinus rhythm, and atrial fibrillation (AF) self-terminating each time within 20 seconds or less. The same results were observed at GP sites in the left atrium following transseptal puncture.

RF energy was applied to sites identified by fractionation mapping and HFS response. The ablation areas included the posterior RA GP, posteromedial RA GP, left anterior GP, left inferior GP, and right anterior GP. RF lesions were delivered with 40 W power, >10 g contact force, and targeting an impedance drop of >10 ohms. At least 1 RF lesion in each of the aforementioned areas resulted in an abrupt HR increase (≥10 BPM), suggestive of perturbation of baseline vagal tone. This included the left anterior GP, discordant with some prior reports where asystole has been observed with ablation at this site, underscoring the overall heterogeneity of cardiac vagal inputs. At the left inferior GP, a greater number of lesions resulted in HR increases than any other GP. Further, the change in baseline HR after all RF applications were delivered at this site was more pronounced than at other GP, increasing from the 75–80 BPM range to the 90–95 BPM range. Throughout the procedure, the baseline HR progressively increased as GP were ablated.

Atropine was again administered at the end of ablation, and the patient’s HR increased from a rate of 100 BPM to only 103 BPM. Postablation conduction intervals were consistent with sinus tachycardia and a shorter atrial-His interval. Telemetry overnight demonstrated baseline HR in the 80–100 BPM range. Of note, she suffered an episode of nausea/vomiting on the ward, a well-documented trigger for her syncope, during which her HR increased to 120–130 BPM, with no evidence of sinus slowing or pauses.

**Figure 2:**
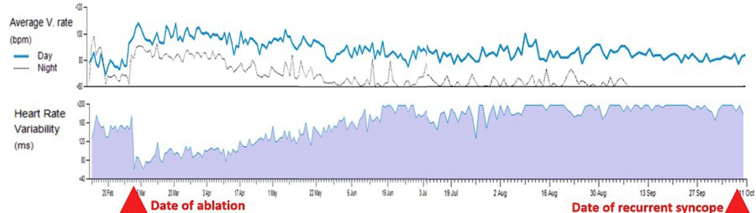
Ventricular rate and heart rate variability (HRV). The patient’s implantable loop recorder (ILR) recorded an abrupt increase in her average ventricular rate and an abrupt decrease in her HRV upon cardioneuroablation. In the months following, the patient’s ILR recorded a gradual regression to preprocedural values in both average ventricular rate and HRV, culminating in her recurrent syncopal event over 7 months later.

In clinic 1 month postprocedure, she reported that she had not experienced recurrent syncope, but she suffered from palpitations, dizziness, shortness of breath, and postural tachycardia, all of which had gradually lessened in the weeks following CNA. Review of her ILR data exhibited abrupt drop-off of her heart rate variability (HRV) and an abrupt increase in her average ventricular rate (Figure 2) following CNA. In clinic 4 months postprocedure, she remained free from syncope, and she reported that her palpitations and postural tachycardia episodes had significantly improved. Her average ventricular rates had followed a gradual trend approaching her preprocedural baseline, as had her HRV.

**Figure 3:**
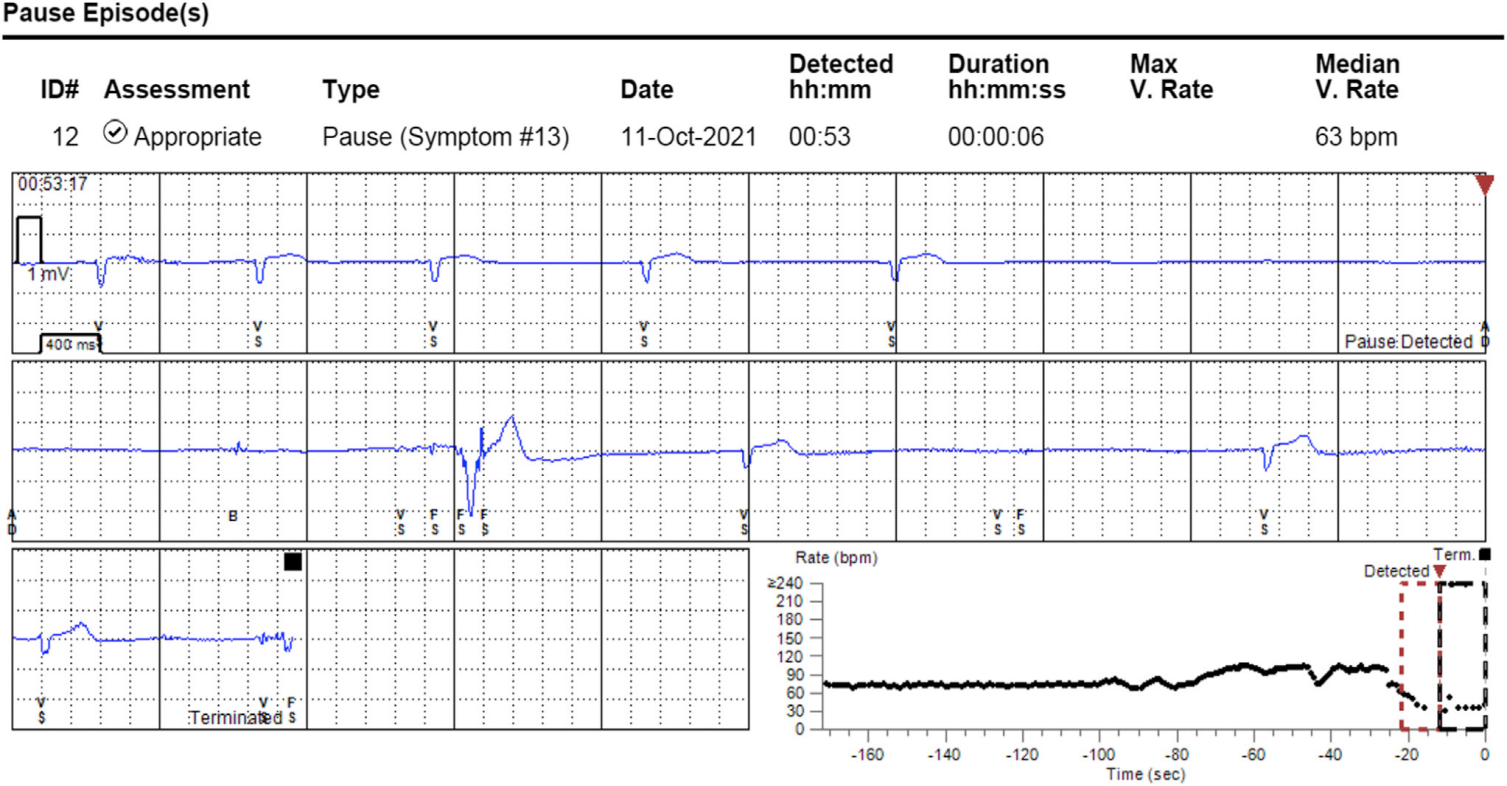
Recurrent syncopal event. The patient’s implantable loop recorder recorded gradual sinus slowing leading to junctional beats, followed by 7 seconds of sinus arrest with asystole, causing syncope.

Seven months postprocedure, she called to report an early morning syncopal episode after she got up to walk to the bathroom at 2:00 AM. A remote transmission from her ILR demonstrated sinus slowing preceding a sinus pause with no ventricular escape rhythm, which correlated with her reported syncopal event (Figure 3).

## Discussion

Establishing parameters, protocols, and meaningful endpoints all represent obstacles in the evolution of novel therapeutic procedures. Fortunately, the rationale for targeting GP inputs in CNA is based on a multifaceted approach (anatomic, electrocardiographic, physiologic, and pharmacologic), which lends objectivity to the procedural protocol such that even novice operators can expect a successful outcome.^12^ In our case, objective data led to an expectation for clinical success postprocedurally, and the patient was syncope-free for 7 months, marking a dramatic improvement from her preprocedural quality of life. Although we did not expose this patient to atropine following her recurrent syncope, which may have further supported the hypothesis that parasympathetic reinnervation was the cause of the return of HRV and syncope, we believe that this remains the most likely explanation.

Our patient’s HRV trend over time is striking (Figure 2). The abrupt drop-off with CNA is followed by a very gradual recovery over the ensuing months, ultimately culminating in her repeat syncopal episode. In fact, this finding that HRV gradually recovered in the months following CNA has been previously described in a separate class of reports, where GPs were targeted to treat AF.^13^ Despite recovery of autonomic function, AF suppression remained durable. The mechanism of this observed paradox is not well understood, a point underscored by our present case, in which our patient achieved short-term autonomic denervation, recovered, and yet failed to maintain the clinical benefit. One explanation for this disparity could be that the mechanism by which autonomic innervation modulates AF differs from that for VVS; however, this would not account for the durable success rates in CNA-treated VVS patients described elsewhere in the literature. Further investigations should be undertaken to elucidate this complex physiologic relationship between acute autonomic denervation, the reinnervation phenomenon, and durability of VVS suppression.

As a nascent procedure, CNA must reckon with protocol and endpoint limitations. Perhaps foremost is the overall lack of precision in mapping GP via both HFS and anatomical models. There is not much allowance for anatomical variation, at times forcing the operator to finish off a CNA via a “learning by burning” approach, monitoring for HR increase after each RF application. Vagal testing has also been problematic. Some laboratories have described exchanging the reliance upon intraprocedural atropine testing for vagal assessment with extracardiac vagal stimulation. Extracardiac vagal stimulation may more accurately distinguish patients who are truly vagally suppressed following CNA and could serve as an additional endpoint for the procedure. In addition, the timing of atropine testing prior to CNA may affect postablation assessment with repeat atropine challenge. Therefore, preprocedural atropine may be given the day before the procedure or during an initial evaluation remote from the CNA.

An additional dilemma in the present case lies in determining the most appropriate next step in management for this patient. Conservative measures have failed to buy her an acceptable quality of life. Although her mother’s case completely resolved with permanent pacing, she is only 20 years old, and implanting a pacemaker may not address a vasomotor component of her VVS. Repeat CNA may result in more durable denervation, though suppressing her vagal tone the first time caused bothersome tachycardia with palpitations, which, if repeated, may require permanent pharmacologic interventions such as beta blockade or ivabradine.

## Conclusion

CNA is a novel and promising therapeutic option for select patients suffering from VVS. The observational literature reports striking success in eliminating syncope in this population, and recurrent syncope post-CNA has not been well described. Herein we report a case of recurrent VVS despite acutely successful CNA 7 months prior, with continuous ILR monitoring documenting evidence of recovery of autonomic innervation. While the present ILR data depict a gradual recovery of GP inputs to the heart, the precise neurobiological mechanism for this patient’s recurrence remains ill-defined and warrants further study.

**Key Teaching Points**

• Cardioneuroablation (CNA) is a novel invasive management option for vasovagal syncope (VVS). It represents a possible alternative to implanting a permanent pacemaker in a young patient population.
• There have been many published reports of CNA in recent years, all reporting low incidences of syncope recurrence following CNA. This is a unique report in its emphasis on corresponding implantable loop recorder data underscoring the physiology and timing of parasympathetic reinnervation.
• This patient’s heart rate variability dropped abruptly with CNA, and then gradually recovered to baseline. This has been described in atrial fibrillation (AF) patients whose ganglionated plexi were ablated; yet those patients maintained AF suppression. It is interesting here that documented “reinnervation” occurred, yet VVS also recurred despite having met adequate procedural parameters initially.
